# Market Assessment and Product Evaluation of Probiotic Containing Dietary Supplements Available in Bangladesh Market

**DOI:** 10.1155/2015/763796

**Published:** 2015-11-16

**Authors:** Anjuman Ara Begum, D. M. Jakaria, Sharif Md Anisuzzaman, Mahfuzul Islam, Siraje Arif Mahmud

**Affiliations:** ^1^Department of Pharmacy, Jahangirnagar University, Savar, Dhaka 1342, Bangladesh; ^2^Department of Biotechnology and Genetic Engineering, Jahangirnagar University, Savar, Dhaka 1342, Bangladesh

## Abstract

Probiotics containing food supplements available in Bangladesh market were identified and collected for assessment. To assess their label claim, they were resuspended into sterile distilled water. Then, series dilutions of each sample solution were prepared and immediately plated out, in duplicate, into De Man Rogosa Sharpe (MRS) agar. These plates were then incubated at 37°C for 48 hours and colonies were counted. Viable cell numbers stated on the labels were compared with actual viable cell numbers. To assess the viability of the probiotics included in the products, probiotic strains were isolated from each of the four products and screened for inhibitory activity against six indicator strains. It was surprisingly found that although the viable cell numbers of all supplements were three to four log cycles lower than label claim of the products, however, this problem did not affect the inhibitory activity of the probiotic strains against indicator strains according to* in vitro* assessment. Legislation and regulation regarding prebiotic-probiotic containing products should be built up in Bangladesh to ensure quality products supply to the consumers. Moreover, manufacturers of probiotic containing products should take the responsibility for providing the consumer with scientifically and legally correct information.

## 1. Introduction

The role of the diet is to provide nutrients to meet host physiological requirements. The concept of functional foods has been evolved recently after extensive research to find out the role of nutrients on health [1]. Functional foods contain physiologically active components, which provide health benefits beyond basic nutrition by affecting one or more functions in the body in a targeted way [2]. “The functional component could include essential macronutrient with specific physiological effect and components that have some nutritive value but are not classified as ‘essential', such as oligosaccharides, or food components with no nutritive value, such as live microorganisms or plant chemicals” [3]. A dietary supplement is a product intended for ingestion as a supplement to the diet and it can be manufactured as pills, tablets, capsules, gel caps, liquids, and powders [4]. Dietary supplements or functional foods contain vitamins, micronutrients, antioxidants, certain bioactive peptides, herbs, polyunsaturated fatty acids, and so forth. The concept has now moved markedly towards gastrointestinal function, in particular the impact of gut bacteria. The most frequently used dietary method of influencing the gut flora composition is that of probiotics. Probiotics are live microorganisms (in most cases, bacteria) that are similar to beneficial microorganisms found in the human gut. According to FAO/WHO (Food and Agriculture Organization and World Health Organization), probiotics are defined as “live microorganisms which when administered in adequate amounts confer a health benefit on the host” [5]. Over 100,000 billion bacteria (more than 500 species) live in the human gut. Human gut flora contains friendly and harmful bacteria. The metabolism products of friendly bacteria (probiotic) such as lactic acid and acetic acid can inhibit the growth of harmful bacteria and confer health benefits on the host [6]. Probiotics are ingested for their purported positive advantages in the digestive tract and/or systemic areas like the liver, vagina, or bloodstream, for example, neutralization of toxins, increase of the immune response [7, 8], antimutagenic and anticarcinogenic activities [9–11], reduction of cholesterol levels [12], control of diarrhea [13], alleviation of lactose intolerance [14], and inflammatory bowel diseases [15]. They are also a source of vitamins, especially of the B group [16, 17], and can also be used as complementary and alternative medicine (CAM) [18]. Consumers should be provided with an independent assessment of physiological, microbial, and safety aspects of these live microbial products, especially if they can improve health. The latest trend in the functional food market is to combine probiotics with prebiotics to enhance the effect of probiotics [19]. Prebiotics are defined as “nondigestible food ingredients that beneficially affect the host by selectively stimulating the growth and/or activity of probiotic bacteria in the colon” [1]. Probiotic trials should use the best methodologies available. For probiotics to exert beneficial properties, they must have a high viability in the product and have robust survival properties in the gut, which is their first point of contact. Because of the presence of potentially pathogenic species such as* Enterococcus faecium* and* E. faecalis* in probiotic products [2, 19], the production and marketing of functional foods should be strictly controlled and carefully monitored [20]. Information on the label of the product, especially regarding the composition and identity of the probiotic strains included, needs to be accurate to guarantee safety and functionality [21]. In most cases, the identity and number of viable strains recovered did not correspond with the information on the label. In view of the above, the aims of this study were to complete a market and product assessment of probiotic containing food supplements available in Bangladesh. This involved identifying the range of products available on the Bangladesh market and evaluating claims made on the labels.

## 2. Methodology

### 2.1. Collection of Sample

To conduct the study, different pharmacies, super shops, and medicine corners were surveyed to find out probiotic containing dietary supplements. Only four different probiotic containing dietary supplements were identified and collected.

### 2.2. Microbial Assessment of Viable Cell Numbers Included in Four Selected Probiotic Supplements

Four probiotic supplements readily available in pharmacies were selected to determine the viability (growth) of the probiotic strains and to compare actual viable cell numbers with the label claim in this regard. The content of the capsule was resuspended in 10 mL sterile distilled water. After performing serial dilution, appropriately diluted solutions were than plated out, in duplicate, onto a De Man Rogosa Sharp (MRS) agar. Plates from each dilution were incubated at 37°C and colonies were counted after 48 hours.

### 2.3. Assessment of the Viability of the Probiotics Available in the Products

To assess the viability of the probiotics included in the products, probiotic strains isolated from each of the 4 products were screened for inhibitory activity against the following 6 indicator strains:* Salmonella typhi*,* Salmonella* sp.,* Shigella* sp.,* Staphylococcus aureus*,* Escherichia coli*, and* Vibrio cholerae*. The probiotic strains were cultured in MR broth (Biolab) for 18 hours at 37°C and 10 *μ*L was spotted on MRS agar (Biolab). The plates were incubated for 24 hours at 37°C and then lawned with active cells of the indicator strains (approximately 10^6^ cfu/mL), embedded in soft agar (0.8%, m/v). The plates were incubated at 37°C for 24 hours and the colonies were examined for the formation of zones, which indicates the level of inhibitory activity and therefore viability. The study was done in triplicate and the averages were determined.

## 3. Results and Discussions

**Figure 1:**
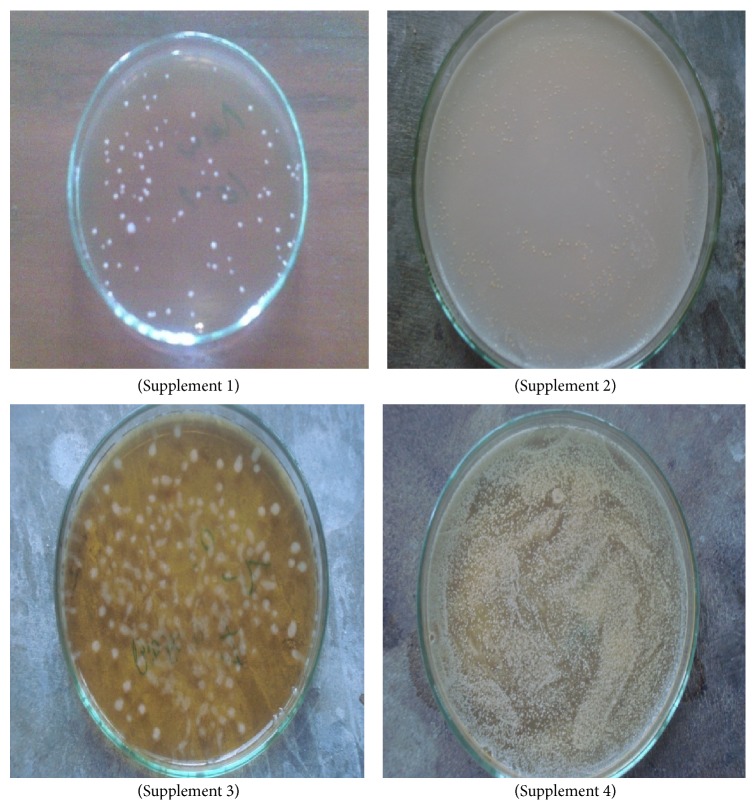
A partial presentation of growth of probiotics of four different products.

The prebiotic and probiotic containing product market is a fast-growing industry worldwide and the list of available products increases on a daily basis. Although as a developing country, Bangladesh has limited scope and facility but is trying to cope up with the health demand of probiotic. One of the leading pharmaceutical companies has realized the importance of functional food and launched a probiotics product in capsule form. Throughout the world prebiotics and probiotics are available in fermented food like yoghurt and also in baby foods. Through the market assessment, different types of food products were found containing probiotics and combination of probiotics and prebiotics. Among them some are fortified baby food, dairy products, and yoghurt products. But only four food supplements were found in Bangladesh containing only probiotics. Among them one product is manufactured in Bangladesh and two capsules were from India and one sachet (powder) was from Taiwan. Three samples were of capsule form and only one was in powdered form available in sachet. But no food supplements were found having probiotic and prebiotic combination. All the four samples contained same probiotics like* Lactobacillus acidophilus*,* Lactobacillus bulgaricus*,* Bifidobacterium bifidum*,* Lactobacillus salivarius*,* Lactobacillus casei*,* Bifidobacterium longum*,* Saccharomyces boulardii*,* Lactobacillus rhamnosus*,* Bifidobacterium infantis*,* Lactococcus lactis*,* Lactobacillus paracasei*, and* Streptococcus thermophilus* but the viable cell numbers are different. As a functional food component, prebiotics and probiotics are conceptually intermediate between foods and drugs. Depending on the jurisdiction, they typically receive an intermediate level of regulatory scrutiny, in particular of the health claims made concerning them. For probiotic to exert beneficial properties, they must have a high viability in the product and have robust survival properties in the gut, which is their first point of contact [22]. That is why assessment of the label claim is necessary for these food products. Comparison of viable cell numbers stated on the labels of the supplements with the actual viable cell numbers is presented in Table 1 and Figure 1.

To achieve health benefits, probiotic bacteria must be viable and available at a high concentration, typically 10^6^–10^7^ cfu/g of product [23]. From this investigation, it was found that viable cell numbers of all supplements were three to four log cycles lower than the claimed number and three of the four products are marginally potential for their proposed health benefit purpose. In different countries, despite the importance of viability, surveys conducted to validate viability claims have shown low populations of probiotic bacteria in probiotic products [24]. Several factors have been claimed to be responsible for the loss of viability of probiotic organisms: acidity of products, acid produced during refrigerated storage (after acidification), level of oxygen in products, oxygen permeation through the package, and sensitivity to antimicrobial substances produced by bacteria [25].

To determine the inhibitory activity of probiotics, six indicator bacterial strains are used. They are* Salmonella typhi*,* Salmonella* sp.,* Shigella* sp.,* Staphylococcus aureus*,* Escherichia coli*, and* Vibrio cholerae*. The result of the screening of the probiotic strains isolated from the 4 selected supplements against 6 indicator strains is presented in Table 2. It is evident that all strains showed good inhibitory activity.

Table 2 reveals that the viable probiotic cells showed good inhibitory activity despite their numbers were lower than respective label claims. Therefore, in order to find out their actual efficacy,* in vivo* assessment should be considered. This situation indicates that as there is no regulation in Bangladesh for these types of products, the consumers are being manipulated by the manufacturers and marketers into buying a product under false claim. It is important that the health claims stated on the labels of products supply the consumer with reliable information because such claims influence consumer behavior and potentially affect public health [26]. Proper manufacturing practice and correct storage should be maintained by the manufacturers and marketers to ensure cell survival.

## 4. Conclusion

Different probiotics containing food supplements and varieties of fortified baby foods (containing probiotics and prebiotics) are imported in Bangladesh. But in Bangladesh there is no regulation to assess the quality of functional food containing products. That is why marketers of these products are misleading consumers with a number of health claims that are not scientifically sound. Now it becomes a prime concern to produce proper legislation and regulation in the field of probiotics and prebiotics containing food supplement and also it should be revised properly, so that quality products of prebiotic-probiotic or functional food can be manufactured and marketed for the benefit of consumers.

## Figures and Tables

**Table 1:** Comparison of the actual viable cell numbers with the claims on the tables of 4 probiotic supplements available in Bangladesh market.

Supplement	Viable cell numbers stated on the label of the supplement (cfu/g)	Actual viable cell numbers identified (cfu/g)
1	4 × 10^9^	3.98 × 10^6^ ± 0.03^*∗*^
2	5 × 10^9^	3.19 × 10^5^ ± 0.5^*∗*^
3	2.5 × 10^9^	1.96 × 10^6^ ± 0.04^*∗*^
4	1 × 10^10^	1 × 10^7^ ± 0.01^*∗*^

^*∗*^Indicates values that are significantly lower than the corresponding viable cell numbers stated on the label of the supplement (*P* < 0.05 after independent sample *t*-test).

**Table 2:** Inhibitory activity of probiotic strains from 4 probiotic supplements against a panel of indicator strains.

Human pathogen	Zone size (mm)			
	Supplement 1	Supplement 2	Supplement 3	Supplement 4
*Salmonella typhi*	26–30	26–31	26–32	26–30
*Salmonella *sp.	31–35	31–33	31-32	31–35
*Shigella *sp.	22–24	22–25	22-23	22–24
*Staphylococcus aureus*	18–22	18–20	18–21	18–22
*Escherichia coli*	24–30	24–30	24–28	24–26
*Vibrio cholerae*	10–14	10-11	10–14	10–13

## References

[1] Gibson G. R. (2007). Functional foods: probiotics and prebiotics. *Culture*.

[2] Roberfroid M. B. (1999). Concepts in functional foods: ش European perspective. *Nutrition Today*.

[3] Brink M., Senekal M., Dicks L. M. T. (2005). Market and product assessment of probiotic/prebiotic—containing functional foods and supplements manufactured in South Africa. *South African Medical Journal*.

[4] Smolin L. A., Grosvenor M. B. (2000). Fat-soluble vitamins and meeting your vitamin needs. *Nutrition Science and Applications*.

[5] Saunier K., Doré J. (2002). Gastrointestinal tract and the elderly: functional foods, gut microflora and healthy ageing. *Digestive and Liver Disease*.

[6] http://www.medicinenet.com/probiotics/article.htm.

[7] Medici M., Vinderola C. G., Perdigón G. (2004). Gut mucosal immunomodulation by probiotic fresh cheese. *International Dairy Journal*.

[8] Ghafoor A., Naseem S., Younus M., Nazir J. (2005). Immunomodulatory effects of multistrain probiotics (Protexin) on broiler chicken vaccinated against avian influenza virus (H9). *International Journal of Poultry Science*.

[9] Boutron-Ruault M.-C. (2007). Probiotiques et cancer colorectal. *Nutrition Clinique et Métabolisme*.

[10] Davis C. D., Milner J. A. (2009). Gastrointestinal microflora, food components and colon cancer prevention. *The Journal of Nutritional Biochemistry*.

[11] Liong M.-T. (2008). Roles of probiotics and prebiotics in colon cancer prevention: postulated mechanisms and in-vivo evidence. *International Journal of Molecular Sciences*.

[12] Zhang M., Hang X., Fan X., Li D., Yang H. (2008). Characterization and selection of *Lactobacillus* strains for their effect on bile tolerance, taurocholate deconjugation and cholesterol removal. *World Journal of Microbiology and Biotechnology*.

[13] Sampalis J., Psaradellis E., Rampakakis E. (2010). Efficacy of BIO K+ CL1285 in the reduction of antibiotic-associated diarrhea—a placebo controlled double-blind randomized, multi-center study. *Archives of Medical Science*.

[14] Guarner F., Perdigon G., Corthier G., Salminen S., Koletzko B., Morelli L. (2005). Should yoghurt cultures be considered probiotic?. *British Journal of Nutrition*.

[15] Kruis W., Frič P., Pokrotnieks J. (2004). Maintaining remission of ulcerative colitis with the probiotic *Escherichia coli* Nissle 1917 is as effective as with standard mesalazine. *Gut*.

[16] Jamaly N., Benjouad A., Bouksaim M. (2011). Probiotic Potential of *Lactobacillus* strains isolated from known popular traditional Moroccan dairy products. *British Microbiology Research Journal*.

[17] Kneifel W., Kaufmann M., Fleischer A., Ulberth F. (1992). Screening of commercially available mesophilic dairy starter cultures: biochemical, sensory, and microbiological properties. *Journal of Dairy Science*.

[18] Saarela M., Mogensen G., Fondén R., Mättö J., Mattila-Sandholm T. (2000). Probiotic bacteria: safety, functional and technological properties. *Journal of Biotechnology*.

[19] Menrad K. (2003). Market and marketing of functional food in Europe. *Journal of Food Engineering*.

[20] Alcid D. V., Troke M., Andszewski S., John J. F. Probiotics as a source of *Enterococcus feacium*.

[21] Roberfroid M. B. (2001). Prebiotics: preferential substrates for specific germs?. *The American Journal of Clinical Nutrition*.

[22] Collins M. D., Gibson G. R. (1999). Probiotics, prebiotics and synbiotics: dietary approaches for the modulation of microbial ecology. *The American Journal of Clinical Nutrition*.

[23] Shah N. P., Ali J. F., Ravula R. R. (2000). Populations of *Lactobacillus acidophilus*, *Bifidobacterium* spp., and *Lactobacillus casei* in commercial fermented milk products. *Bioscience and Microflora*.

[24] Shah N. P., Lankaputhra W. E. V., Britz M. L., Kyle W. S. A. (1995). Survival of Lactobacillus acidophilus and Bifidobacterium bifidum in commercial yoghurt during refrigerated storage. *International Dairy Journal*.

[25] Dave R. I., Shah N. P. (1997). Viability of yoghurt and probiotic bacteria in yoghurts made from commerical starter cultures. *International Dairy Journal*.

[26] Clydesdale F. M. A. (1997). Proposal for the establishment of scientific criteria for health claims for functional foods. *Nutrition Reviews*.

